# Author Correction: Dopamine-dependent prefrontal reactivations explain long-term benefit of fear extinction

**DOI:** 10.1038/s41467-019-08399-4

**Published:** 2019-01-23

**Authors:** A. M. V. Gerlicher, O. Tüscher, R. Kalisch

**Affiliations:** 1 Neuroimaging Center (NIC), Focus Program Translational Neuroscience (FTN), Johannes Gutenberg University Medical Center, Langenbeckstr. 1, 55131 Mainz, Germany; 2 Deutsches Resilienz Zentrum (DRZ), Johannes Gutenberg University Medical Center, Untere Zahlbacher Str. 8, 55131 Mainz, Germany; 3 Department of Psychiatry and Psychotherapy, Johannes Gutenberg University Medical Center, Untere Zahlbacher Str. 8, 55131 Mainz, Germany; 4 Present Address: Department of Clinical Psychology, University of Amsterdam, Nieuwe Achtergracht 129B, 1018 WS Amsterdam, The Netherlands

Correction to: *Nature Communications*; 10.1038/s41467-018-06785-y published online 16 Oct 2018.

In the original version of this Article, the fourth sentence of the legend to Figure 1b incorrectly read “Note, that the group difference stemmed from significantly smaller CS+ evoked SCRs averaged across the whole test phase, but the speed of re-extinction did not differ significantly between drug groups (control analysis with stimulus (CS+, CS−) and trial (1–10) as within-, and group (placebo, L-DOPA) as between-subject factor: stimulus × group, *F*_1,297_ = 6.57, *P* *=* 0.02, partial *η*^2^ = 0.17; stimulus × trial × group, *F*_1,297_ = 1.32, *P* *=* 0.23; *n* = 35)”. The correct version states “*F*_1,33_=6.58” instead of “*F*_1,297_ = 6.57”, and “*F*_9,297_ = 1.32” instead of “*F*_1,297_ = 1.32”. These errors have now been corrected in both the PDF and HTML versions of the Article. The correct values were used in the statistical analysis and the errors do not affect the conclusions.

