# Supplementary material for: Dopamine-dependent prefrontal reactivations explain long-term benefit of fear extinction
Source: Nat Commun. 2018 Oct 16;9:4294. doi: 10.1038/s41467-018-06785-y (PMC6191435; doi:10.1038/s41467-018-06785-y)
Supplement: Supplementary file 1 — Supplementary Information [file 41467_2018_6785_MOESM1_ESM.pdf]

Supplementary Table 1. Statistical comparisons (two-sample *t* tests) of questionnaire scores, body-mass index, US intensity and US rating between placebo and L-DOPA treated groups. Placebo and L-DOPA treated participants did not differ significantly on any measure (all *Ps*>.05; two-sided tests).

	Placebo<br>N = 20<br>mean (SD)	L-DOPA<br>N = 20<br>mean (SD)	*t* -value	*P*-value
STAI-T	32.9 (5.8)	36.5 (6.7)	1.77	.08
ASI-3	14.1 (7.9)	14.5 (5.4)	.16	.87
STAI-S Day 1	32.2 (5.8)	33.5 (5.8)	.69	.49
STAI-S Day 2 pre	32.2 (5.6)	36.5 (7.4)	2.07	.05
STAI-S Day 2 post	30.5 (5.0)	32.2 (6.1)	.97	.34
STAI-S Day 3	32.6 (7.5)	34.1 (8.0)	.59	.56
BMI	24.7 (2.2)	26.4 (4.5)	1.51	.14
US intensity (mA)	15.1 (7.7)	13.1 (7.3)	-.86	.39
US rating (0-10)	6.9 (1.7)	6.7 (1.5)	-.35	.73

STAI-T: trait anxiety; STAI-S: state anxiety; ASI-3: anxiety-sensitivity index; BMI: body-mass index, US rating on a scale from 0-10 ("I do not feel anything"-"strongest pain imaginable delivered by such an electrode").

Supplementary Table 2. The results of the analysis of the SCR during extinction learning on day 2, do not hinge on the exact choice of trials employed for assessing CR at the beginning or end of extinction. That is, when operationalizing CR at the beginning of extinction as CS+/CS- evoked SCR averaged across the first 5 trials (as in the fMRI analysis) instead of the first 20% of trials (i.e. 3 trials) as in all SCR analyses (see Methods) we still find a significant effect of stimulus that does not significantly differ between groups. Similarly, operationalizing CR at the end of extinction as CS+/CS- evoked SCR averaged across the last 5 trials (as in the fMRI analysis), we also find a significant effect of stimulus that does not differ between groups.

	Main effect stimulus	Main effect group	Interaction effect stimulus x group
CR at beginning of ext. (mean CS+/CS- across first 5 trials)	$F_{1,34} = 49.65$<br>$P < .001$<br>partial $\eta^2 = .59$	$F_{1,34} = .36$<br>$P = .55$	$F_{1,34} = .37$<br>$P = .55$
CR at the end of ext. (mean CS+/CS- across last 5 trials)	$F_{1,34} = 23.65$<br>$P<.001$<br>partial $\eta^2 = .41$	$F_{1,34} = .12$<br>$P = .73$	$F_{1,34} = .08$<br>$P = .78$

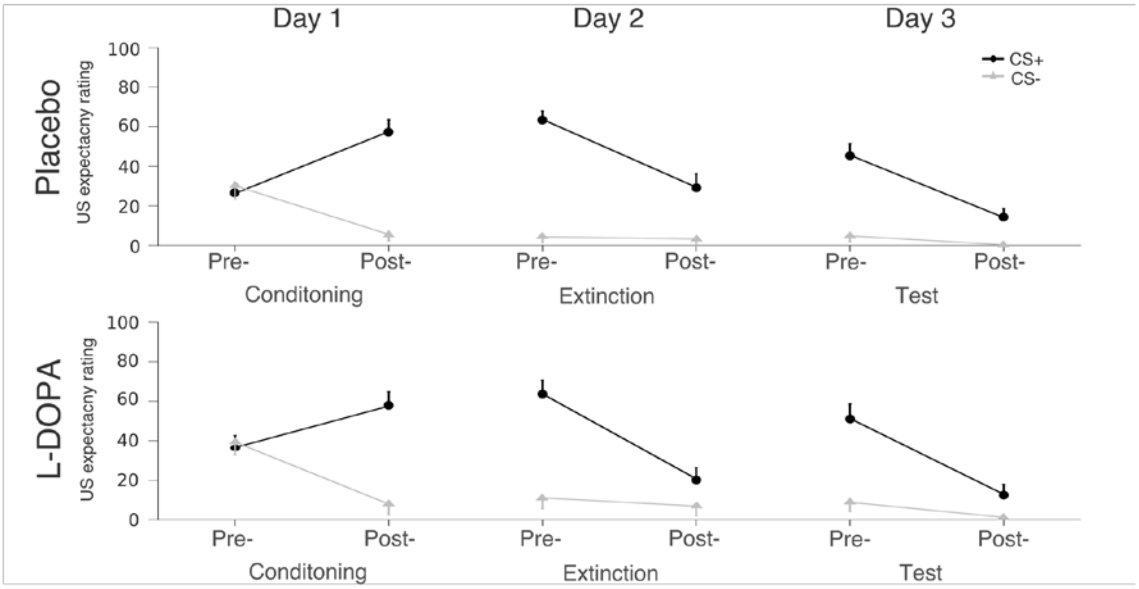

Supplementary Figure 1 | Average US expectancy ratings for CS+ and CS- collected before and after each experimental phase. The post-conditioning US expectancy ratings reflected successful acquisition of CS-US contingencies on day 1 in both groups (repeated-measures ANOVA, stimulus: $F_{1,33}$=75.58, P<.001, partial η^2=.70; stimulus x group: P=.98; n=35). Before the start of the extinction session on day 2 US expectancy ratings were significantly greater for CS+ than CS-, indicating successful recall of CS-US contingencies in both groups (stimulus: $F_{1,34}$=78.06, P<.001, partial η^2=.70; stimulus x group: P=.60; n=36). After the extinction session on day 2 participants on average still showed greater US expectancies for the CS+ than CS- in both groups (stimulus: $F_{1,34}$=16.69, P<.001, partial η^2=.33; stimulus x group: P=.21; n=36). There were significant effects of stimulus before ($F_{1,36}$=56.36, P<.001, partial η^2=.61) and after test on day 3 ($F_{1,36}$=14.47, P=.001, partial η^2=.29; n=38) that did not differ between the placebo and the L-DOPA group (stimulus x group: Ps>.75). This suggests L-DOPA does not induce any change in explicit contingency knowledge, as has been shown in the case of manipulations of memory reconsolidation following reactivation with propranolol[1–3]. Note that sample sizes used for statistical analysis differ slightly across days as not all participants confirmed their response with a button press within the 15 seconds response time window. Data presented as mean ± s.e.m.

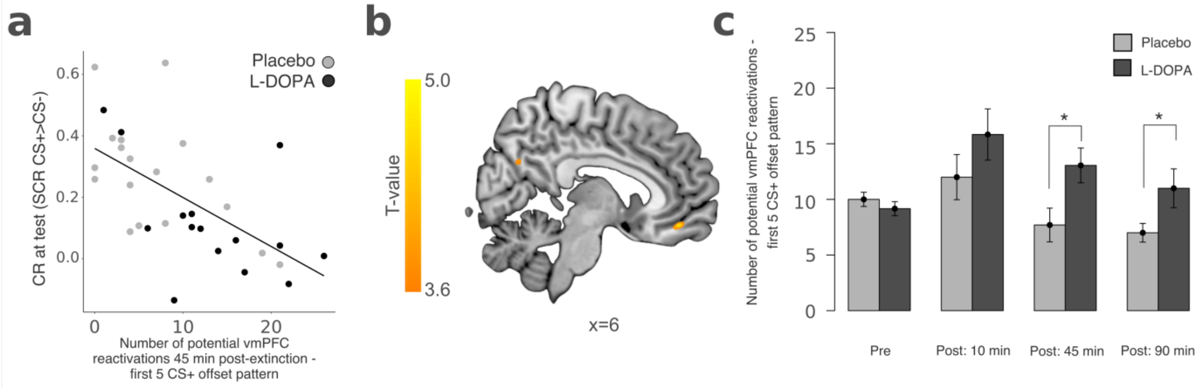

Supplementary Figure 2 | The effects are stable against changes in data transformation. For statistical analysis the number of potential spontaneous vmPFC pattern reactivations were log-transformed in order to account for non-normality of the distribution. To test the robustness of our results, we repeated the analyses on non-transformed data after excluding two outliers (both L-DOPA treated participants; outlier definition: number of reactivations > mean+2 SD). **(a)** When using non-transformed data and excluding the two outliers, the prediction of CR at test on day 3 based on the number of potential spontaneous CS+ offset-related vmPFC reactivations 45 min after extinction on day 2 remained robust (linear regression: β=-.02; *P*=.0002; *n*=35). Including drug group and its interaction with number of reactivations into the regression model did not change the results (multiple linear regression: β_{react}=-.09; *P*=.03; *n*=35) and showed that the relationship between number of reactivations and CR at test did not differ between groups ($\beta_{group*react}$=-.08; *P*=.28). **(b)** The non-transformed number of potential CS+ offset-related vmPFC pattern reactivations also predicted vmPFC activity (CS+>CS-) during test (SPM multiple linear regression: x,y,z=6,44,-14; *Z*=4.29, *P*=.002; SVC, FWE; *n*=40). Display threshold *P*<.05, SVC, FWE corrected, no masking applied. **(c)** The effect of L-DOPA on the number of potential vmPFC pattern reactivations remained robust (repeated-measures ANOVA, time x group: $F_{3,114}$=5.32, *P*=.03, partial η^2=.13; post-hoc *t* tests: pre: *P*=.36; post: 10 min: *P*=.22; 45 min: T_{36}=-2.46, *P*=.02, Cohen's *d*=.80; 90 min: T_{36}=-2.07, *P*=.05, Cohen's *d*=.68; *n*=40). Importantly, using non-transformed data did not change the mediation of the effect of L-DOPA on CR at test by the number of potential reactivations (mediation analysis: β=-.09, 95% CI: -.15--.02, *P*=.008; *n*=35). Alternatively, when applying robust regression to the non-transformed data without preceding outlier exclusion, the prediction of CR at test remained robust (robust linear regression: β_{react}=-.01; *P*=.04; *n*=35). This effect was stable when including group and the group x reactivations interaction (robust multiple linear regression: $\beta_{group*react}$=-.02; *P*=.01; *n*=35), but was then indicated to be weaker in the L-DOPA group ($\beta_{group*react}$=.02; *P*=.02; *n*=35). Note, that this was the only analysis that indicated the presence of a group difference that can be explained by the two outliers in the L-DOPA group. The mediation of the effect of L-DOPA on CR at test by the number of potential vmPFC pattern reactivations, however, remained robust (robust mediation analysis: β=-.06, 95% CI: -.12--.06, *P*=.03; *n*=35). Data are presented as mean ± s.e.m.

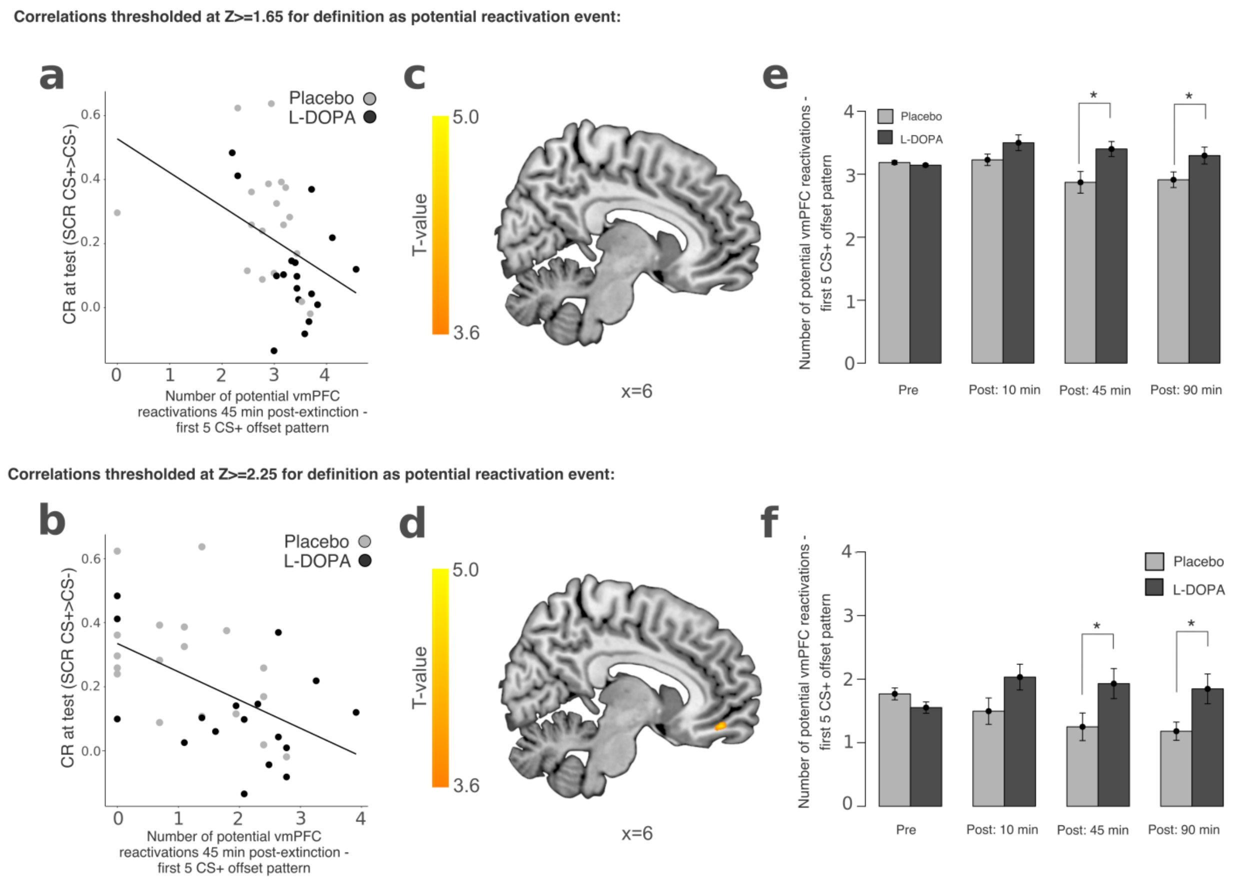

Supplementary Figure 3 | The effects are stable against changes in the threshold used for defining potential spontaneous CS+ offset related vmPFC pattern reactivations. To confirm that the results did not depend on the exact threshold employed for defining a reactivation event (Z>2; as in[4]), we repeated the analyses employing a more liberal (Z>1.65 ~ P<.10; upper panel), and a more conservative threshold (Z>2.25 ~ P<.025; lower panel). Importantly, the prediction of CR (SCR CS+>CS-) at test on day 3 based on potential spontaneous CS+ offset-related vmPFC pattern reactivations was robust against **(a)** defining post-extinction reactivations based on a more liberal threshold (i.e. Z>1.65; linear regression: β=-.11, P=.01; n=35) and **(b)** a more conservative threshold (i.e. Z>2.25; linear regression: β=-.09 P=.001; n=35). Including drug group and its interaction with the number of reactivations into the regression model reduced the effect of the more liberally (multiple linear regression: with Z>1.65: β_{react}=-.05; P=.30; n=35), but not the more conservatively defined reactivations (multiple linear regression: with Z>2.25: β_{react}=-.09; P=.035; n=35) and indicated that the effect did not differ between groups (with Z>1.65: $\beta_{group*react}$=-.07; P=.45; with Z>2.25: $\beta_{group*react}$=-.03; P=.58). **(c)** Similarly, the prediction of vmPFC activity (CS+>CS-) during test on day 3 based on potential vmPFC pattern reactivations was less robust against using a more liberal threshold (with Z>1.65; SPM multiple linear regression: x,y,z=6,48,-12; Z=3.27, P=.06; SVC, FWE; n=40), than **(d)** a more conservative threshold (with Z>2.25; SPM multiple linear regression: x,y,z=6,46,-14; Z=4.15, P=.004; SVC, FWE; n=40) for the pattern reactivations. Display threshold P<.05, SVC, FWE corrected, no masking applied. The exact threshold definition did also not affect the effect of L-DOPA on the number of potential vmPFC pattern reactivations, see **(e)** for results with a more liberal (repeated-measures ANOVA, time x group: $F_{3,114}$=3.10, P=.04, partial η^2=.08, Greenhouse-Geisser corrected; post-hoc two-sample t tests: pre: P=.28; post: 10 min: P=.09; 45 min: T_{38}=-2.53, P=.02, Cohen's d=.80; 90 min: T_{38}=-2.10, P=.04, Cohen's d=.68; n=40) and **(f)** for results with a more conservative threshold (repeated-measures ANOVA, time x group: $F_{3,114}$=3.37, P=.02, partial η^2=.08; post-hoc two-sample t tests: pre: P=.10; post: 10 min: P=.07;

45 min: T_{38}=-2.12, P=.04, Cohen's d=.67; 90 min: T_{38}=-2.42, P=.02, Cohen's d=.77; n=40). In addition, the exact threshold definition did not affect the mediation of the effect of L-DOPA on CR at test by the number of potential spontaneous CS+ offset-related vmPFC pattern reactivations using a more conservative threshold (with Z>2.25; mediation analysis: β=-.06, 95% CI: -.12--.01, P=.04; n=35), even though this effect was only a near-significant trend when using the more liberal threshold (with Z>1.65; mediation analysis: β=-.05, 95% CI: -.10--.00, P=.06; n=35). Data are presented as mean ± s.e.m.

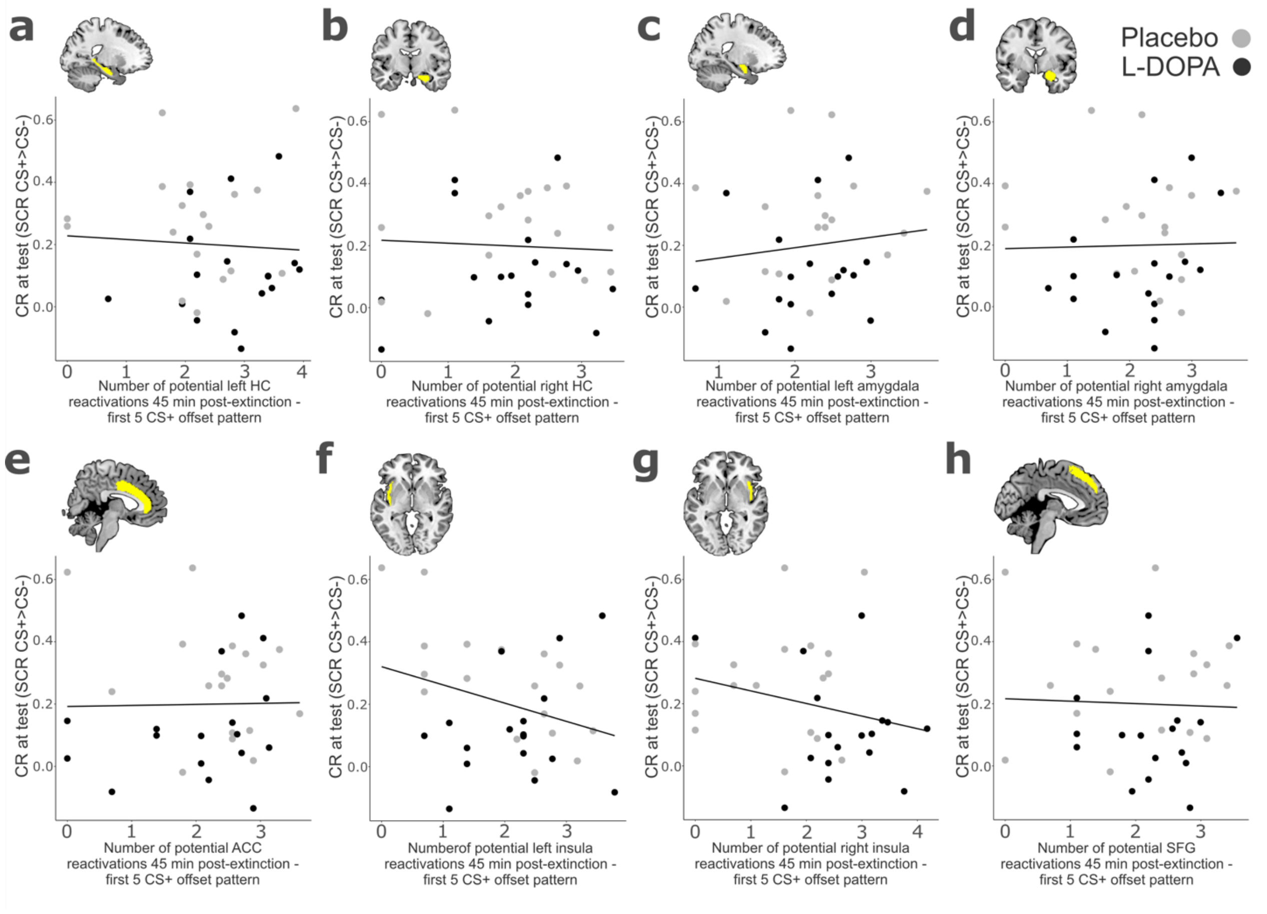

Supplementary Figure 4 | Potential spontaneous CS+ offset-related pattern reactivations 45 min after extinction on day 2 in control regions do not predict CR (SCR CS+>CS-) at test on day 3. The number of potential CS+ offset-related pattern reactivations did not predict CR at test (linear regression; *n*=35) in the **(a)** left hippocampus (HC; *P*=.73), **(b)** right hippocampus (HC; *P*=.77), **(c)** left amygdala (*P*=.58), **(d)** right amygdala (*P*=.89), **(e)** anterior cingulate cortex (ACC; *P*=.92), **(f)** left insula (*P*=.08), **(g)** right insula (*P*=.16) or **(h)** the superior frontal gyrus (SFG; *P*=.82). When including those regions' number of reactivations, together with the number of reactivations in the vmPFC, into a regression model, vmPFC reactivations remained the only significant predictor of CR at test on day 3 (multiple linear regression: β=-.10, *P*=.006; all other *P*s>.30; *n*=35). No region showed any significant relationship between potential reactivations during rest 10 or 90 minutes after extinction and CR at test. Following previous research, all regions of interest were selected from the Harvard-Oxford Atlas (see Online Methods) and thresholded at 50% tissue probability as illustrated by inlets.

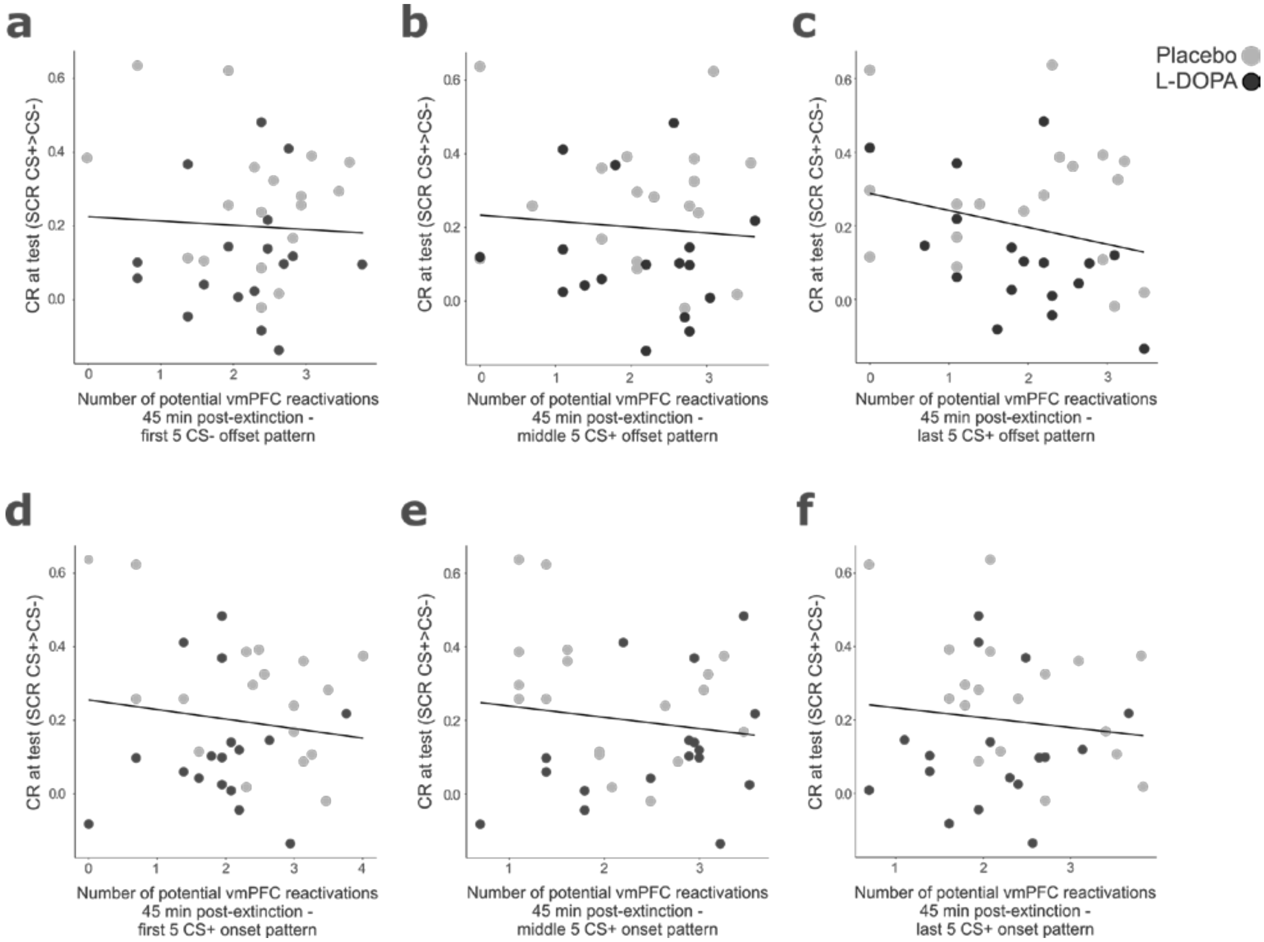

Supplementary Figure 5 | The effects are specific to reactivations of the CS+ offset-related vmPFC activity pattern early in the extinction session. (a) The number of potential spontaneous reactivations (45 min post-extinction) of the CS- offset related vmPFC pattern (i.e. first 5 CS- offsets for which US omission is expected by the participant) does not predict CR at test (linear regression: β_{45min}=-.02, SE=.04, T_{33}=-.30, *P*=.76; *n*=35). There was also no relationship between spontaneous CS- offset-related vmPFC reactivations at 10 or 90 minutes after extinction learning and CR at test (data not shown, *Ps*>.14). Similarly, there was no relationship between the number of potential spontaneous reactivations (45 min post-extinction) of the pattern elicited by the CS+ offsets during **(b)** the middle (i.e. 6th -10th trial; linear regression: β_{45min}=-.02 *P*=.63; *n*=35) and **(c)** the end of the extinction session (11th -15th trial; linear regression: β_{45min}=-.05, *P*=.15; *n*=35) (i.e. where repeated US omission has been experienced by the participant) and CR at test. Spontaneous CS+ offset-related vmPFC reactivations from the middle or end of extinction learning reactivation at 10 or 90 minutes after extinction learning were also not related to CR at test (data not shown, *Ps*>.15). **(d)** Critically**,** the effects are also not observed for reactivations (45 min post-extinction) of the first 5 CS+ *on*set-related vmPFC activity pattern (linear regression: $ß_{45min}$=-.03, SE=.03, T_{33}=-.79, *P.*=43; *n*=35). There was also no relationship between spontaneous reactivations of the first 5 CS+ *on*set-related vmPFC activity pattern at 10 or 90 minutes after extinction learning and CR at test (data not shown, *Ps*>.07). **(e)** Similarly, the effects are also not observed for reactivations (45 min post-extinction) of the middle 5 CS+ *on*set-related vmPFC activity pattern (linear regression: $ß_{45min}$=-.03, SE=.04, T_{33}=-.81, *P*=.42; *n*=35). There was no relationship between spontaneous middle 5 CS+ *onset*-related vmPFC reactivations at 10 or 90 minutes after extinction learning and CR at test (data not shown, *Ps*>.13). **(f)** Finally, the effects are also not observed for reactivations (45 min post-extinction) of the last 5 CS+ *on*set-related vmPFC activity pattern

(linear regression: $ß_{45min}$=-.03, SE=.04, T_{33}=-.66, *P*=.51; *n*=35). There was no relationship between spontaneous last 5 CS+ *onset*-related vmPFC reactivations at 10 or 90 minutes after extinction learning and CR at test (data not shown, *Ps*>.12).

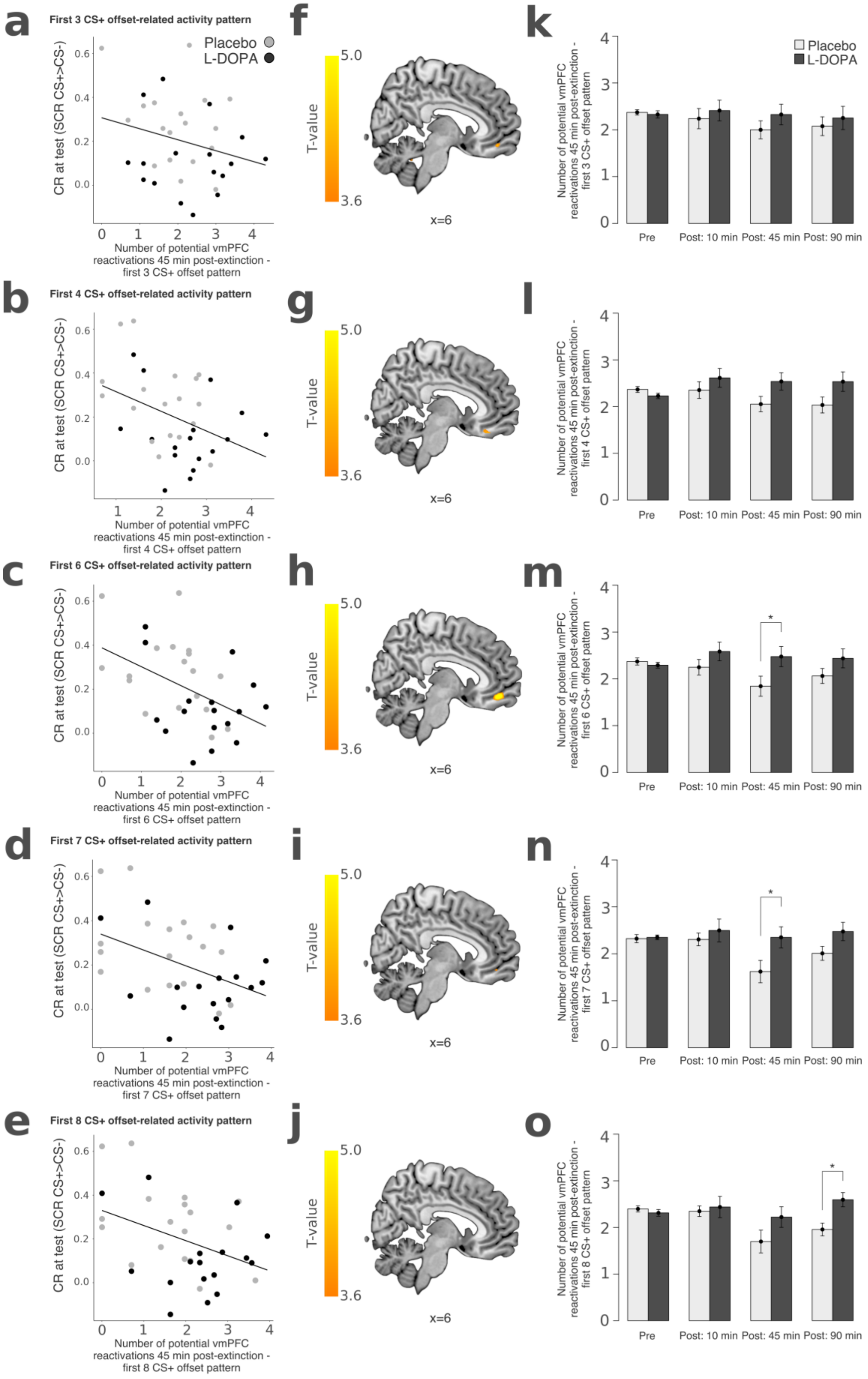
a
First 3 CS+ offset-related activity pattern
Placebo
L-DOPA
CR at test (SCR CS+>CS-)
Number of potential vmPFC reactivations 45 min post-extinction - first 3 CS+ offset pattern
b
First 4 CS+ offset-related activity pattern
Number of potential vmPFC reactivations 45 min post-extinction - first 4 CS+ offset pattern
c
First 6 CS+ offset-related activity pattern
Number of potential vmPFC reactivations 45 min post-extinction - first 6 CS+ offset pattern
d
First 7 CS+ offset-related activity pattern
Number of potential vmPFC reactivations 45 min post-extinction - first 7 CS+ offset pattern
e
First 8 CS+ offset-related activity pattern
Number of potential vmPFC reactivations 45 min post-extinction - first 8 CS+ offset pattern
f
g
h
i
j
T-value
5.0
3.6
x=6
k
l
m
n
o
Pre
Post: 10 min
Post: 45 min
Post: 90 min
Number of potential vmPFC reactivations 45 min post-extinction - first 3 CS+ offset pattern
Number of potential vmPFC reactivations 45 min post-extinction - first 4 CS+ offset pattern
Number of potential vmPFC reactivations 45 min post-extinction - first 6 CS+ offset pattern
Number of potential vmPFC reactivations 45 min post-extinction - first 7 CS+ offset pattern
Number of potential vmPFC reactivations 45 min post-extinction - first 8 CS+ offset pattern

Supplementary Figure 6. | The effects are stable against changes in the number of trials employed for estimating the CS+ offset-related vmPFC activity pattern from early extinction. For the main analysis we selected the vmPFC activity pattern evoked by the first 5 CS+ offsets early during extinction. To test the robustness of our results against changes in the exact number of trials used for estimating the CS+ offset-related vmPFC activity pattern, we repeated all analyses based on the first 3 to 8 CS+ offsets. The prediction of CR at test (SCR CS+>CS-) on day 3 was not significant when defining the vmPFC activity pattern based on **a)** the first 3 CS+ offsets (β=-.05, P=.14; n=35). However, the prediction of CR at test (SCR CS+>CS-) on day 3 remained robust when defining the vmPFC activity pattern based on **b)** the first 4 CS+ offsets (β=-.08, P=.02; n=35), **c)** the first 6 CS+ offsets (β=-.09, P=.005; n=35), **d)** the first 7 CS+ offsets (β=-.07, P=.009; n=35) and **e)** the first 8 CS+ offsets (β=-.07, P=.02; n=35). Including drug group and its interaction with CS+ offset related vmPFC reativations into the regression model did not change the results and did not reveal any difference in the relationship between vmPFC reactivations and CR at test between placebo and L-DOPA treated participants, as in the main analysis (all Ps>.42). Recruitment of the vmPFC during test (CS+>CS-) could also be predicted based on spontaneous reactivations of **f)** the first 3 CS+ offsets (x,y,z=6,46,-14; Z=4.02, P=.006, SVC, FWE; n=40), **g)** the first 4 CS+ offsets (x,y,z=4,34,-20; Z=3.61, P=.02, SVC, FWE; n=40), **h)** the first 6 CS+ offsets (x,y,z=6,46,-14; Z=5.34, P<.001, SVC, FWE; n=40), and **i)** the first 7 CS+ offsets (x,y,z=8,46,-14; Z=3.90, P=.009, SVC, FWE; n=40), but only a near-significant trend for **j)** the first 8 CS+ offsets (x,y,z=6,44,-14; Z=3.27, P=.061, SVC, FWE; n=40). All display thresholds P<.05, SVC, FWE corrected, no masking applied. **k)** There was no significant effect of L-DOPA on number of vmPFC activity pattern reactivations computed based on the first 3 CS+ offsets (repeated-measures ANOVA, time x group: $F_{3,114}$=.48, P=.70; n=40). **l)** There was a near-significant trend towards a greater number of vmPFC activity pattern reactivations computed based on the first 4 CS+ offsets after L-DOPA intake (repeated-measures ANOVA, time x group: $F_{3,114}$=2.30, P=.08; n=40) specifically 45 min after extinction (post-hoc two-sample t tests: pre: P=.12; post: 10 min: P=.34; 45 min: T_{38}=-1.95, P=.06; 90 min: T_{38}=-1.86, P=.07; n=40). **m)** There was a near-significant main effect of drug group independent of time on the first 6 CS+ offsets (repeated-measures ANOVA, group: $F_{1,38}$=3.66, P=.06; n=40) due to significantly more vmPFC reactivations 45 min after extinction in L-DOPA treated participants (post-hoc two-sample t tests: pre: P=.42; post: 10 min: P=.20; 45 min: T_{38}=-2.09, P=.04, Cohen's d=.67; 90 min: T_{38}=-1.46, P=.15; n=40). **n)** The number of reactivations of the first 7CS+ offset vmPFC activity pattern was significantly greater in the L-DOPA group (repeated-measures ANOVA, group: $F_{1,38}$=4.39, P=.04, partial η^2=.07; n=40), due to an effect of L-DOPA on number of vmPFC reactivations 45 min after extinction (post-hoc two-sample t tests: pre: P=.78; post: 10 min: P=.50; 45 min: T_{38}=-2.32, P=.03, Cohen's d=.71; 90 min: T_{38}=-1.90, P=.06; n=40). **o)** Lastly, on the first 8 CS+ offsets there was a near-significant time by group interaction (repeated-measures ANOVA, time x group: $F_{3,114}$=3.10, P=.06, Greenhouse-Geisser corrected) with L-DOPA treated participants showing significantly more vmPFC reactivations specifically 90 min after extinction (post-hoc two-sample t tests: pre: P=.39; post: 10 min: P=.73; 45 min: P=.12; 90 min: T_{38}=-3.11, P=.004, Cohen's d=.97; n=40). Note, that the effects are not robust to repeating the analyses with vmPFC activity patterns evoked by less than 3 or more than 8 CS+ offsets.

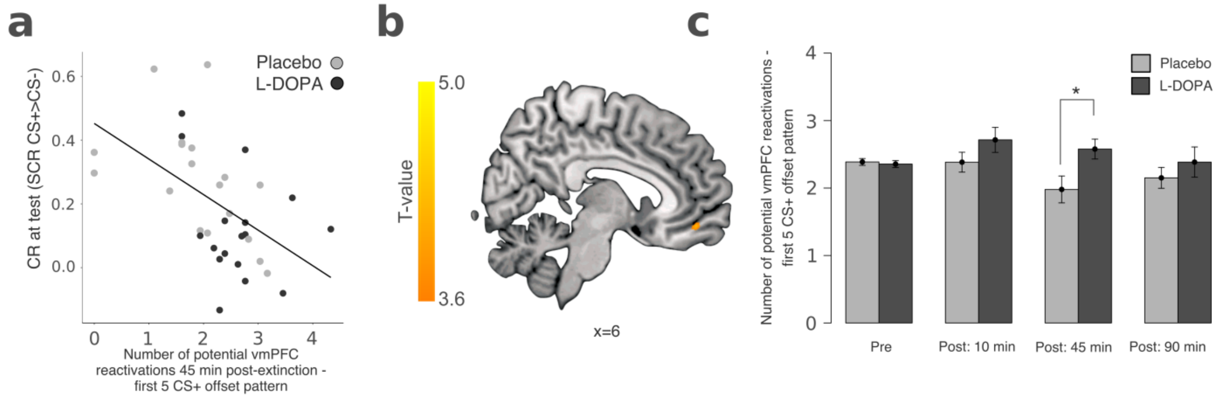

Supplementary Figure 7 | Regressing out nuisance signals from the resting-state time courses before the reactivation analysis does not change the results. The resting-state data were analyzed in accordance with a previous study[4] that did not explicitly control for the influence of spontaneous fluctuations in physiological signals of no interest or head motion. In order to test whether our results were affected by such nuisance signals we repeated the analysis on resting-state time courses cleaned from mean cerebrospinal fluid, mean white matter and the six head motion signals using the 'regress out covariate' function provided by the Resting-State fMRI Data Analysis Toolkit[5] (REST). **(a)** The prediction of CR at test on day 3 based on potential spontaneous CS+ offset-related vmPFC reactivations (45 min) after extinction remained robust (linear regression: β=-.11, *P*=.001; *n*=35). Including drug group and its interaction with the number of reactivations into the regression model did not change the effect (multiple linear regression: β_{react}=-.10; *P*=.03) and indicated that the effect did not differ significantly between groups ($\beta_{group*react}$=-.03, *P*=.69; *n=35*). **(b)** Similarly, the prediction of vmPFC activity (CS+>CS-) during test on day 3 based on potential vmPFC pattern reactivations remained robust (SPM multiple linear regression: x,y,z=6,48,-12; *Z*=3.84, *P*=.01; SVC, FWE; *n*=40). Display threshold *P*<.05, SVC, FWE corrected, no masking applied. **(c)** There was a near-significant trend towards a main effect of drug group on number of CS+ offset-related vmPFC reactivations (repeated-measures ANOVA, group: $F_{1,38}$=3.99, *P*=.05, partial η^2=.10; *n*=40), due to significantly greater number of reactivations in L-DOPA compared to placebo treated participants 45 min after extinction (two-sample *T*-test: post-hoc *t* tests: pre: *P*=.66; post: 10 min: *P*=.17; 45 min: T_{36}=-2.42, *P*=.02, Cohen's *d*=.77; 90 min: *P*=.40; *n*=40). In addition, the mediation of the effect of L-DOPA on CR at test by the number of potential spontaneous CS+ offset-related vmPFC pattern reactivations remained robust (indirect mediation effect: β=-.06, 95% CI: -.11--.02, *P*=.01; *n*=35).

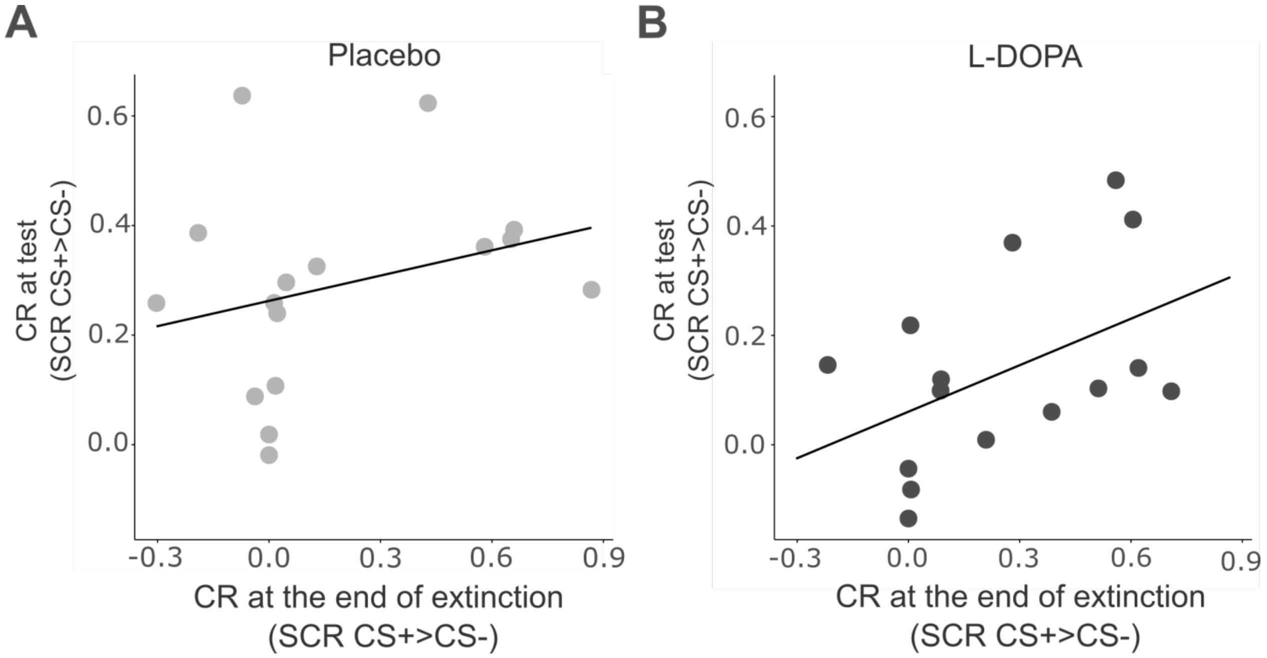

Supplementary Figure 8 | Relationship between CRs at the end of extinction and CRs at test. In order to identify the best possible behavioral predictor of extinction memory retrieval, we tested the prediction of CRs at test based on drug treatment (placebo/L-DOPA) as well as either i) the relative reduction of CRs from the beginning to the end of extinction (mean difference between SCRs to CS+>CS- to the first vs. the last 20% of trials) or ii) CRs at the end of extinction (SCRs to CS+>CS- to last 20% of trials), using multiple linear regression. The relative reduction of CRs from the beginning to the end of extinction on day 2 did not predict CRs at test on day 3 (β=.00, SE=.10, T_{30}=.01, *P*=.99; *n*=33). There was a weak, but significant positive relationship between CRs (SCR CS+>CS-) at the end of extinction learning on day 2 and CRs (SCR CS+>CS-) at test on day 3 (β=.21, SE=.10, T_{30}=2.19, *P*=.04). Including the interaction between group and CRs at the end of extinction into the model indicated that the relationship did not differ significantly between groups (interaction: β=.13, SE=.20, T_{29}=.68, *P*=.50). Note, that analysis of simple slopes in each group indicated, though, a near-significant trend towards a positive relationship between CRs at the end of extinction and CRs at test after L-DOPA ($\beta_{L\text{-}DOPA}$=.29, SE=.15, T_{29}=1.93, *P*=.08), but not after placebo administration ($\beta_{placebo}$=.15, SE=.13, T_{29}=1.17, *P*=.25). That is, in line with recent studies on other pharmacological extinction enhancers[6], the relationship between CRs at the end of extinction and CRs at test may be stronger after enhancing extinction memory consolidation pharmacologically using L-DOPA. The results do not change when controlling for inter-individual differences in initial fear acquisition (last 20% of SCRs to CS+>CS on day 1) and fear memory recall (first 20% of SCRs to CS+>CS- trials on day 2; data not shown).

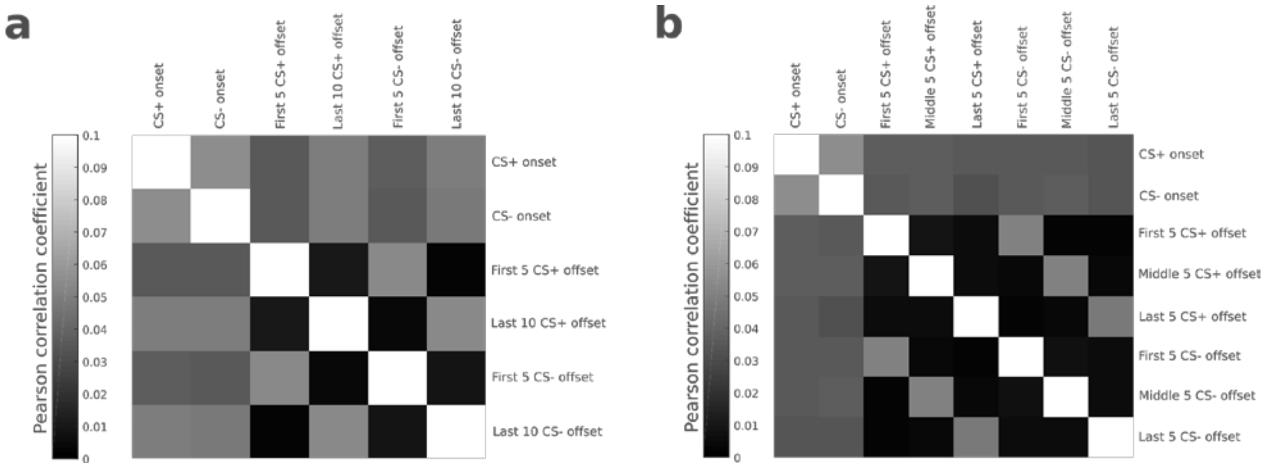

Supplementary Figure 9 | Pearson correlation coefficients between CS on- and offset related regressors in the general linear models (GLM) used for the main and the control analysis. The short interval between CS on- and offset of only 4.5 sec raises the possibility that the CS+ offset-related regressor used for the reactivation analysis may be affected by high collinearity with the CS+ onset-related regressor. **a)** However**,** in the GLM used for the main analysis the Pearson correlation coefficients between all regressors convolved with the hemodynamic response function (HRF) were low on average. Importantly, the regressor corresponding to the first 5 CS+ offsets was not correlated with the regressor corresponding to the CS+ onsets (R=.04, P>.05; n=40). **b)** Similarly**,** in the GLM used for the control analysis the regressors corresponding to the first, middle or last 5 CS+ offsets were not correlated with the regressor corresponding to the CS+ onset (all Rs<.04, Ps>.05; n=40).

References

1. Kindt, M., Soeter, M. & Vervliet, B. Beyond extinction: erasing human fear responses and preventing the return of fear. *Nat. Neurosci.* **12,** 256–258 (2009).
2. Soeter, M. & Kindt, M. Dissociating response systems: Erasing fear from memory. *Neurobiol. Learn. Mem.* **94,** 30–41 (2010).
3. Soeter, M. & Kindt, M. Noradrenergic enhancement of associative fear memory in humans. *Neurobiol. Learn. Mem.* **96,** 263–271 (2011).
4. Staresina, B. P., Alink, A., Kriegeskorte, N. & Henson, R. N. Awake reactivation predicts memory in humans. *Proc. Natl. Acad. Sci. U. S. A.* **110,** 21159–21164 (2013).
5. Song, X.-W. *et al.* REST: a toolkit for resting-state functional magnetic resonance imaging data processing. *PloS One* **6,** e25031 (2011).
6. Otto, M. W. *et al.* Enhancement of Psychosocial Treatment With D-Cycloserine: Models, Moderators, and Future Directions. *Biol. Psychiatry* **80,** 274–283 (2016).
